# Radioactive Iodide (^131^I^−^) Excretion Profiles in Response to Potassium Iodide (KI) and Ammonium Perchlorate (NH_4_ClO_4_) Prophylaxis

**DOI:** 10.3390/ijerph9082936

**Published:** 2012-08-16

**Authors:** Curtis Harris, Cham Dallas, Edward Rollor, Catherine White, Benjamin Blount, Liza Valentin-Blasini, Jeffrey Fisher

**Affiliations:** 1 Institute for Health Management and Mass Destruction Defense, College of Public Health, University of Georgia, Athens, GA 30603, USA; Email: cdallas@ihmd.uga.edu (C.D.); erollor@ihmd.uga.edu (E.R.); cwhite@rx.uga.edu (C.W.); 2 National Center for Environmental Health, Division of Laboratory Sciences, Centers for Disease Control and Prevention, Atlanta, GA 30341, USA; Email: bkb3@cdc.gov (B.B.); lbv5@cdc.gov‎ (L.V.-B.); 3 National Center for Toxicological Research, U.S. Food and Drug Administration, Jefferson, AR 72079, USA; Email: jeffrey.fisher@fda.hhs.gov (J.F.)

**Keywords:** perchlorate, iodide, radioiodide, thyroid

## Abstract

Radioactive iodide (^131^I^−^) protection studies have focused primarily on the thyroid gland and disturbances in the hypothalamic-pituitary-thyroid axis. The objective of the current study was to establish ^131^I^−^ urinary excretion profiles for saline, and the thyroid protectants, potassium iodide (KI) and ammonium perchlorate over a 75 hour time-course. Rats were administered ^131^I^−^ and 3 hours later dosed with either saline, 30 mg/kg of NH_4_ClO_4_ or 30 mg/kg of KI. Urinalysis of the first 36 hours of the time-course revealed that NH_4_ClO_4_ treated animals excreted significantly more ^131^I^−^ compared with KI and saline treatments. A second study followed the same protocol, but thyroxine (T_4_) was administered daily over a 3 day period. During the first 6–12 hour after ^131^I^−^ dosing, rats administered NH_4_ClO_4_ excreted significantly more ^131^I^−^ than the other treatment groups. T_4_ treatment resulted in increased retention of radioiodide in the thyroid gland 75 hour after ^131^I^−^ administration. We speculate that the T_4_ treatment related reduction in serum TSH caused a decrease synthesis and secretion of thyroid hormones resulting in greater residual radioiodide in the thyroid gland. Our findings suggest that ammonium perchlorate treatment accelerates the elimination rate of radioiodide within the first 24 to 36 hours and thus may be more effective at reducing harmful exposure to ^131^I^−^ compared to KI treatment for repeated dosing situations. Repeated dosing studies are needed to compare the effectiveness of these treatments to reduce the radioactive iodide burden of the thyroid gland.

## 1. Introduction

Stable iodide (^127^I^−^), as potassium iodide (KI) or “dietary iodine”, has been recognized for over 30 years as a practical thyroid radioprotectant for people exposed to radioactive isotopes of iodide [1,2]. In a recent National Academies of Science report on the distribution and administration of KI in the event of a nuclear incident [3], sodium perchlorate (Irenat^®^) was recommended for adults if KI treatment was contraindicated, such as in patients with preexisting thyroid disease. Successful thyroid radioprotectant treatments, such as KI or ammonium perchlorate (NH_4_ClO_4_), are judged by their ability to block or limit thyroidal uptake of trace amounts of radioactive iodide (e.g., ^131^I^−^), while limited research has focused on treatment doses promoting excretion of the isotopes. Currently, the United States Food and Drug Administration (US FDA) recommends KI tablets as a preventative treatment for ^131^I^−^ poisoning of the thyroid gland [4]. 

Studies utilizing rats in the 1950s and 1960s demonstrated that perchlorate (ClO_4_^−^) altered the serum pharmacokinetics of ^131^I^−^ [5,6,7]. The kinetic profile of ^131^I^−^ in laboratory animals treated with ClO_4_^−^ was characterized by decreased serum ^131^I^−^ levels and increased urine ^131^I^−^ levels [6]. In the presence of ClO_4_^−^, ^131^I^−^ is excreted in urine more rapidly, presumably because perchlorate blocks basolateral and/or apical uptake of ^131^I^−^ into sodium iodide symporter- or pendrin-protein rich tissues, such as the thyroid and small intestine [5,8,9,10,11,12]. Additionally, perchlorate administration displaces non-organified iodide from the thyroid, and the perchlorate discharge test has been used to diagnose iodide organification defects in humans for decades [10]. Perchlorate has been shown to have side effects ranging from rash and fever to several fatal cases of aplastic anemia [13,14], though these effects typically manifest from large bolus doses administered over weeks or months [15]. 

Rats administered ClO_4_^−^ intravenously (0.1–3.0 mg/kg) excreted approximately 83% of the administered ClO_4_^−^ dose over 24 hours [16]. In a similar experiment, these authors also intravenously dosed rats with 3.3 mg/kg of isotonically labeled ClO_4_^−^ (^36^ClO_4_) and reported that 96% of the dose was excreted in 24 hours and 99.5% by 48 hours [17]. In a recent study, an oral dose of 30 mg/kg of ClO_4_^−^ was administered to rats, and 38% of the dose was recovered in urine within the first 12 hours after dosing [15]. 

In the present rat study, the effectiveness of KI and NH_4_ClO_4_ to increase excretion of ^131^I^−^ into urine over a 3 day period was assessed, with and without T_4_ administration. In previous rat studies we reported that over a 15 hour post dosing period, KI and NH_4_ClO_4_ were equally effective at reducing thyroid gland exposure to ^131^I^−^ [15]. However, NH_4_ClO_4_ was more effective at increasing urinary clearance of ^131^I^−^ than KI. This study extends the timeframe of the experiment from 15 to 75 hours and also evaluates the influence of T_4_ administration on ^131^I^−^ kinetics. Rats, unlike humans, have short thyroxine half-lives in serum, and are sensitive to up-regulation of the thyroid gland by thyroid stimulating hormone (TSH) [18]. To control the potential for TSH mediated stimulation of the thyroid gland, one subset of rats for each treatment group received replacement doses of T_4_ to suppress the potential for TSH release [19,20].

## 2. Materials and Methods

### 2.1. Chemicals

Ammonium perchlorate (99.8%), 100% ethanol, and sodium hydroxide were purchased from Aldrich (Milwaukee, WI, USA). Nonradioactive thyroxine was purchased from Sigma Chemical Corporation (St. Louis, MO, USA). Potassium iodide was obtained from J. T. Baker (Phillipsburg, NJ, USA), carrier-free iodide-131 (^131^I^−^) from Amersham Biosciences (29.4 mCi/µg), acepromazine maleate (10 mg/mL) and ketamine HCl (100 mg/mL) from Fort Dodge Animal Health (Fort Dodge, IA, USA) and xylazine (20 mg/mL) from Ben Venue Laboratories (Bedford, OH, USA). Isoflurane (99.9%) was purchased from Abbott Laboratories (Abbott Park, IL, USA). 

### 2.2. Animals and Experimental Design

Male Sprague-Dawley rats (330 ± 30 g, approximately 11 weeks old) from Harlan Laboratories (Indianapolis, IN, USA) were provided LabDiet™ Laboratory Rodent Diet 5001 rat chow and water *ad libitum*. The animals used in this study were handled in accordance with the procedures of The University of Georgia Institutional Animal Care and Use Committee (IACUC), AUP# A2005-10110-0. The rats experienced a 12 hour light and dark cycle, with room air temperature at 22 ± 2 °C and relative humidity at 50 ± 20%. 

Rats were housed individually in metabolism cages for a 5 day acclimation period prior to the start of the experiments. Twelve hours before the experiment commenced, food was removed from the animals to ensure complete absorption of the radiotracer and treatment doses. A summary of the experiments is shown in Table 1. The general experimental protocol was to dose rats orally with 2.91 µCi (6 ng/kg) ^131^I^−^ in saline solution (1 mL) by oral gavage and then return the rats to their metabolism cages. Rats from Group 1 (n = 6 for each treatment group) were removed from their metabolism cages after 3 hours and dosed by oral gavage with 1 mL of either 0.9% saline, 30 mg/kg of KI (calculated as iodide) or 30 mg/kg of NH_4_ClO_4_ (calculated as ClO_4_^−^) dissolved in 0.9% aqueous saline. Rats from Group 2 (n = 6 for each treatment group) followed the same experimental protocol as Group 1 except each animal received a 0.1 mL intraperitoneal (ip) injection of 15 µg/kg of T_4_ (based on euthyroid replacement T_4_ doses administered by [21]) dissolved in 0.1 M NaOH and 1 animal from each treatment group received a 0.1 mL ip injection of 0.1 M NaOH (controls). Data from animals in Group 2 that received ip injections of NaOH had not statistically different from animals of a similar treatment in Group 1. As a result, data from animals in Group 2 that received ip injections of NaOH were assimilated with animals of a similar treatment in Group 1. The rats were held in metabolism cages for 75 hours for urine collections. Urine from Groups 1 and 2 were collected via metabolism cage vials at +3, 6, 12, 18, 24, 30, 36, 42, 48, 54, 60, 66, 72, and 75 hours. Blood was collected from the tail vein of animals in dose Groups 1 and 2 at 15 hours after dosing with ^131^I^−^ and at sacrifice. At sacrifice (75 hours after dosing with ^131^I^−^), animals in Groups 1 and 2 were anesthetized with a ketamine cocktail (50 mg/kg ketamine, 3.3 mg/kg xylazine, and 3.4 mg/kg acepromazine administered at 0.1 mL per 100 g BW), then killed by asphyxiation at +75 hours. Blood was collected via cardiac puncture and serum prepared by centrifugation at 1,500 rpm at 4 °C for 15 min. Thyroid lobes were removed from the trachea and weighed. Urine was removed from the bladder via syringe. Sera, urine, and thyroid glands were stored at −80 °C until analysis.

**Table 1:** Summary of experiments in the rat to characterize excretion profiles of ^131^I^−^ following prophylactic administration of saline, KI, or perchlorate with and without T_4_ hormone replacement.

Experiment	Description	Dose (mg/kg)	Urine Collection Times (hours)	T_4_ Injection Times (hours)	Serum and Thyroid Collection Time (hours)
**Group 1** (n = 36) 12 rats each for saline, KI and NH_4_ClO_4_ treatment groups	Single oral dose of ^131^I^−^ followed 3 hours later by single oral doses of saline, KI, or NH_4_ClO_4_	ORAL ^131^I^−^: 6 × 10^−6^ KI: 30 ClO_4_: 30	3, 6, 12, 18, 24, 30, 36, 42, 48, 54, 60, 66, 72, and 75	---	Serum: 15 and 75 Thyroid: 75
**Group 2** (n = 18) 6 rats each for saline, KI and NH_4_ClO_4_ treatment groups	Equal to Group 1	Equal to Group 1 ip T_4_: 0.015	Equal to Group 1	T_4_ doses at time of saline, KI and NH_4_ClO_4_ doses, then 24, and 48 hours later	Serum: 15 and 75 Thyroid: 75

### 2.3. ^131^I^−^ Analysis

Serial urine samples were placed on a gamma counter (1470 Wallac Wizard) equipped with one detector and ^131^I^−^ counts/minute (cpm) were measured within 2 hours after collection. ^131^I^−^ cpm were also assessed in whole thyroids and serum samples within 2 hours of sacrifice. Raw counts were recorded. Urine and sera were then stored at −80 °C for no less than 80 days (10 half-lives for ^131^I^−^) in order for the radioactivity to decay.

Serum TSH measurements were made using a rat TSH radioimmunoassay kit from A. F. Parlow and the National Hormone & Peptide Program (lot numbers AFP329691Rb, AFP11542B, and AFP5512B).

### 2.4. ^127^I^−^ and ClO_4_^−^ Analysis

Non-radioactive analytes (^127^I^−^ and ClO_4_^−^) were quantified using ion chromatography coupled with tandem mass spectrometry. Serum samples were spiked with internal standard (^129^I^−^ and Cl^18^O_4_^−^), treated to remove proteins, and analyzed by ion chromatography electrospray ionization tandem mass spectrometry [22]. Urine samples were spiked with internal standard (^129^I^−^ and Cl^18^O_4_^−^) and analyzed by ion chromatography electrospray ionization tandem mass spectrometry [23]. 

### 2.5. Urinary Excretion Kinetics of Anions

All urine samples for Groups 1 and 2 were analyzed for ^131^I^−^ excretion kinetics. Animals in Groups 1 and 2 that received ^127^I^−^ or ClO_4_^−^ also had urine samples analyzed for ^127^I^−^ or ClO_4_^−^ excretion kinetics. All half-life calculations were prepared using Win Non Lin 5.2 software. 

### 2.6. Statistical Analysis

Single factor analysis of variance (ANOVA) was used initially to determine significance across the treatment groups (control saline, KI and NH_4_ClO_4_) with statistical significance set at *p* < 0.05. Once statistical significance was determined across the treatment groups by ANOVA, a limited number of comparisons were carried out using a two-sample t-test (assuming equal variance) to compare each treatment group (*p* < 0.05) to control and to each other. All calculations were performed using Microsoft Excel. It should be noted that animals in Group 2 that received ip injections of NaOH were lumped together with animals in Group 1 of a similar treatment dose, *i.e.*, saline, KI, or ClO_4_^−^.

## 3. Results

### 3.1. ^131^I^−^ Excretion in Urine

**Figure 1:**
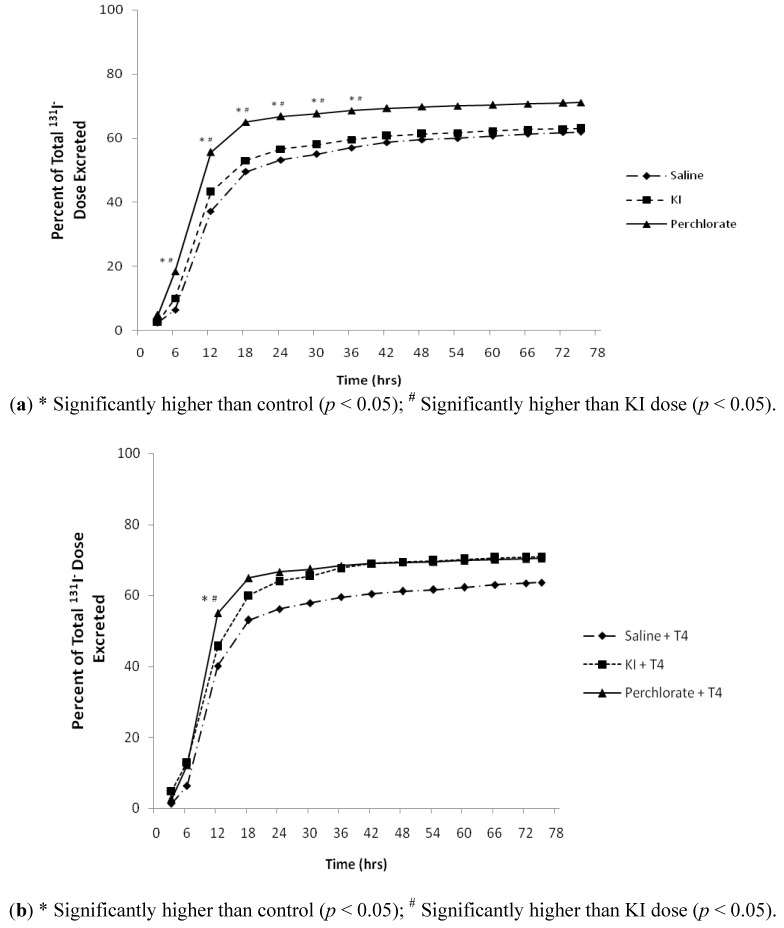
(**a**) Percent of total ^131^I^−^ dose excreted in urine 75 hour of Group 1 male rats collected via metabolism cages dosed with ^131^I^−^ followed by saline, KI (30 mg/kg), or perchlorate (30 mg/kg) 3 hours later (n = 12), statistical analysis was conducted on concentration data for each individual time-point (data not shown); (**b**) Percent of total ^131^I^−^ dose excreted in 75 hour urine of Group 2 male rats collected via metabolism cages dosed with ^131^I^−^ followed by saline, KI (30 mg/kg), or perchlorate (30 mg/kg) at +3 hours and dosed with replacement T_4_ at +3, +27, and +51 hours (n = 6), statistical analysis was conducted on concentration data for each individual time-point (data not shown).

Rats were administered ^131^I^−^, then 3 hours later either saline, 30 mg/kg of KI or 30 mg/kg of NH_4_ClO_4_ by oral bolus gavage. Group 2 was distinguished from Group 1 (Table 1) by repeated T_4_ administration, first at the time of dosing with saline, KI or NH_4_ClO_4_, then at +27 hours and +51 hours during the time-course. The 3-day cumulative volumes of urine produced were 39.7 ± 7.7 mL for Group 1 and 41.3 ± 5.7 mL for Group 2. In Group 1, 71, 63, and 62% of the administered ^131^I^−^ doses for NH_4_ClO_4_, KI, and control saline treatment groups, respectively (Figure 1a), were excreted in urine by 75 hours after dosing with ^131^I^−^. In Group 2, 72, 71, and 63% of the administered doses of ^131^I^−^ were excreted in urine for the NH_4_ClO_4_, KI, and saline control treatment groups, respectively (Figure 1b) by 75 hours after dosing with ^131^I^−^. Most of the ^131^I^−^ collected over the 3-day period (60.5 ± 6%) was excreted in urine by 24 hours after dosing in all treatments for Groups 1 and 2 (Figure 1(a,b)). The 24-hour urinary excretion half-lives for ^131^I^−^ (Table 2) in control and KI treated rats ranged from 3.5 to 4 hours; conversely, NH_4_ClO_4_ treated rats excreted ^131^I^−^ more rapidly (2.6 hour urinary excretion half-life, *p* < 0.001). 

**Table 2:** Twenty four hour urinary excretion half-lives for ^131^I^−^ for Group 1 and 2 rats.

^131^I^−^ Excretion Half Lives (hours)		
	Group 1 ^131^I^−^	Group 2 ^131^I^−^ + T_4_
Saline Control	4.0 ± 0.7	3.7 ± 1.2
KI	3.5 ± 0.5	3.8 ± 0.3
NH_4_ClO_4_	2.6 ± 0.4 ^*,#^	2.6 ± 0.4 ^*,#^

Rats for each Group were orally dosed with ^131^I^−^ and then 3 hours later orally dosed with saline, KI (30 mg/kg), or NH_4_ClO_4_ (30 mg/kg). Group 2 rats also received ip injections of thyroxine (0.015 mg/kg) 3 hours following ^131^I^−^ administration and then every 24 hours until the conclusion of the experiment. Urine for each Group was collected for 24 hours; * statistically significantly lower than saline treatment (*p* < 0.001); ^#^ statistically significantly lower than KI treatment (*p* < 0.001).

### 3.2. ^131^I^−^ in Serum and Thyroid

**Figure 2:**
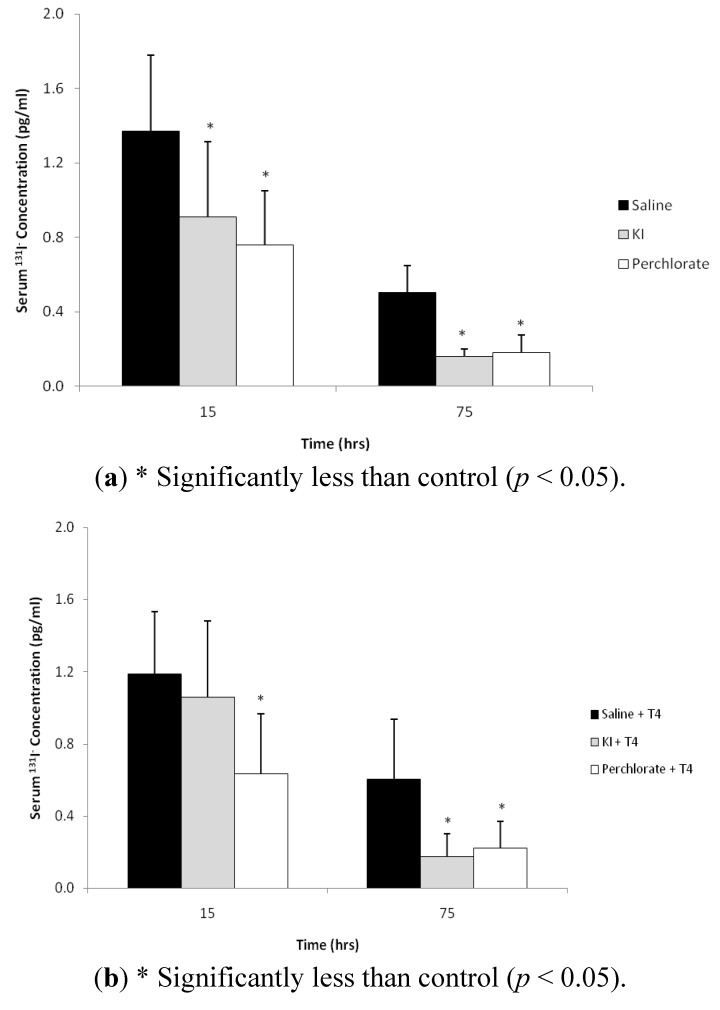
(**a**) ^131^I^−^ concentrations (pg/mL) in serum of Group 1 male rats collected via tail vein bleed at 15 and via cardiac puncture 75 hours after oral administration of ^131^I^−^ and 12 and 72 hours after oral administration of either saline, KI (30 mg/kg), or perchlorate (30 mg/kg) as described in Figure 1a. Data are means ± standard deviation (n = 12); (**b**) ^131^I^−^concentrations (pg/mL) in serum of Group 2 male rats collected via tail vein bleed at +15 hours and via cardiac puncture at 75 hours after oral administration of ^131^I^−^. Animals were dosed with ^131^I^−^ followed by saline, KI (30 mg/kg), or perchlorate (30 mg/kg) and dosed with replacement T_4_ as described in Figure 1b. Data are means ± standard deviation (n = 6).

The mean ^131^I^−^ concentration in the Group 1 control serum at 15 hours after ^131^I^−^ dosing was 1.37 ± 0.41 pg/mL and decreased to 0.50 ± 0.15 pg/mL at 75 hours post dosing (Figure 2a). In Group 1, the mean ^131^I^−^ serum levels in the KI and the NH_4_ClO_4_ treatment groups at 15 hours were 0.91 ± 0.40 and 0.76 ± 0.29 pg/mL, and decreased to 0.16 ± 0.04 and 0.18 ± 0.09 pg/mL, respectively, at 75 hours post dosing. The ^131^I^−^ serum concentrations in the KI and NH_4_ClO_4_ treatment groups were significantly less than saline controls for both sampling times (*p* < 0.05). The addition of T_4_ proved to have little effect on mean serum ^131^I^−^ concentration for Group 2 (Figure 2b). The mean serum ^131^I^−^ concentrations at 15 hours following T_4_ and saline, KI and NH_4_ClO_4_ treatments was 1.2 ± 0.35, 1.06 ± 0.42, and 0.64 ± 0.33 pg/mL respectively, and decreased to 0.61 ± 0.33, 0.17 ± 0.12, and 0.22 ± 0.15 pg/mL at 75 hours post dosing. At 15 hours post ^131^I^−^ dosing, only the NH_4_ClO_4_ treatment group ^131^I^−^ concentrations were significantly less (*p* < 0.05) than controls, while both KI and NH_4_ClO_4_ treatment group ^131^I^−^ concentrations were significantly less than controls at the 75 hour sampling time.

**Figure 3:**
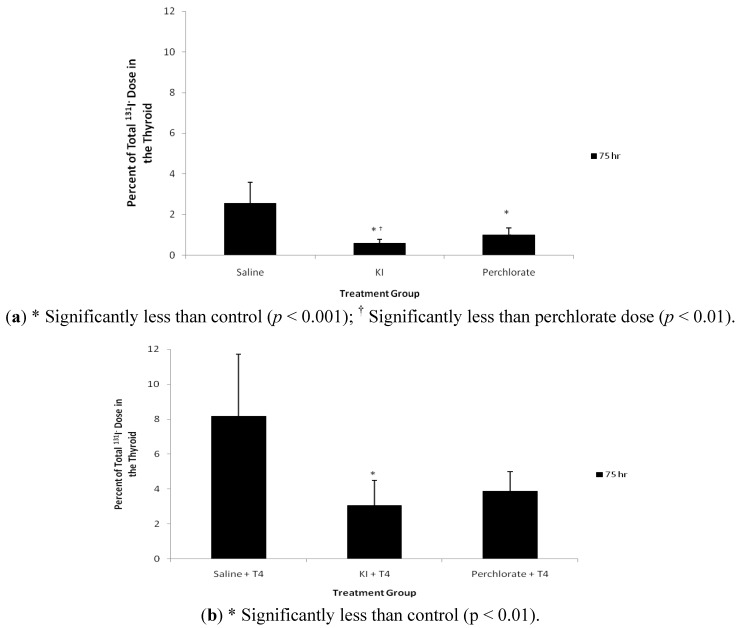
(**a**) 75 hour percent of total ^131^I^−^ in the thyroid of Group 1 male rats dosed with ^131^I^−^ via gavage followed by saline, KI (30 mg/kg), or perchlorate (30 mg/kg) as described in Figure 1a. Data are means ± standard deviation (n = 12); (**b**) 75 hour + T_4_ percent of total ^131^I^−^ in the thyroid of Group 2 male rats dosed with ^131^I^−^ via gavage followed by saline, KI (30 mg/kg), or perchlorate (30 mg/kg) and dosed with T_4_ replacement as described in Figure 1b. Data are means ± standard deviation (n = 6).

**Figure 4:**
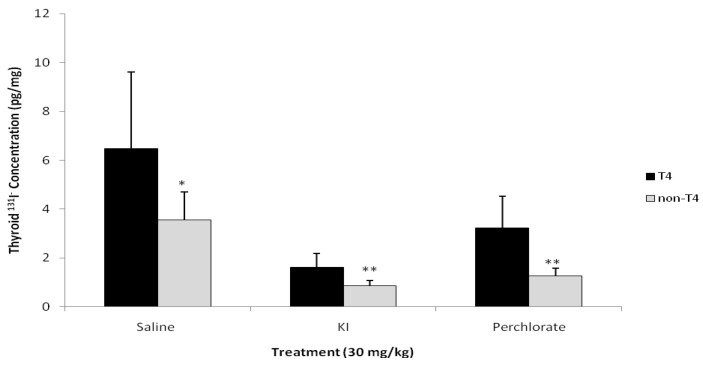
Comparison of ^131^I^−^ concentration in the thyroid of male rats administered T_4_ *versus* rats not administered hormone replacement therapy (n = 12 for non-T_4_ and n = 6 for T_4_). * Saline treatment without T_4_ has a significantly less ^131^I^−^ thyroid concentration than saline with T_4_ (*p* < 0.05); ** KI and perchlorate treatment without T_4_ has a significantly less ^131^I^−^ thyroid concentration than KI and perchlorate with T_4_ (*p* < 0.001).

Compared with control saline, KI and NH_4_ClO_4_ treatment reduced levels of ^131^I^−^ in thyroid gland at 75 hours post exposure (Figure 3a). Also the residual ^131^I^−^ levels in the thyroid gland in the KI treatment group were lower than the NH_4_ClO_4_ treatment group (*p* < 0.01). KI and NH_4_ClO_4_ treatment reduced the thyroid content of ^131^I^−^ by 77 and 61%, respectively, 3 days after administration of ^131^I^−^. Group 2 animals treated with T_4_ displayed a different thyroidal ^131^I^−^ content (Figure 3b). The mean residual percentage of ^131^I^−^ doses were less in both the KI (38% of control) and NH_4_ClO_4_ (48% of control) treatment groups, compared with saline controls; however, only the KI treatment was significantly less than controls (*p* < 0.01). Interestingly, control, KI, and NH_4_ClO_4_ treated rats from Group 2 retained more thyroidal ^131^I^−^ than rats from Group 1, which did not receive T_4_ treatment. Figure 4 compares the thyroidal ^131^I^−^ concentrations for Groups 1 and 2. In all cases, T_4_ treatment resulted in increased thyroidal ^131^I^−^ concentrations (*p* < 0.05). 

### 3.3. ^127^I^−^ and ClO_4_^−^: Urinary Excretion and Serum Concentrations

In Group 1 rats, the cumulative amounts of ^127^I^−^ and ClO_4_^−^ excreted in urine over 72 hours were 128 and 92%, respectively, of the administered doses of ^127^I^−^ as KI, and ClO_4_^−^, as NH_4_ClO_4_. Most of the excretion of ^127^I^−^ (93%) and ClO_4_^−^ (97%) occurred within 24 hours of dosing. In the T_4_ treated group, 106% and 86% of ^127^I^−^ and ClO_4_^−^, respectively, was excreted in urine by 72 hours after dosing. Eighty four and 96% of the excreted ^127^I^−^ and ClO_4_^−^ anions occurred within 24 hours.

Stable iodide urinary half lives determined using 24 h urine collections in rats that were treated with KI for Groups 1 and 2 were 3.5 ± 0.6 and 3.9 ± 0.3 hours respectively, with no significance between groups. Perchlorate 24 hour urinary half lives in rats that were treated with NH_4_ClO_4_ for Groups 1 and 2 were 2.5 ± 0.5 and 2.5 ± 0.4 hours, respectively. 

Serum concentrations of ^127^I^−^ and ClO_4_^−^ 72 hours after administration were 200.4 ± 199.8 and 81.3 ± 31.6 ng/mL, respectively, in Group 1 and 56.7 ± 15.9 ng/mL and 26.3 ± 14.6 ng/mL, respectively in Group 2.

### 3.4. Serum TSH Levels

The mean serum TSH concentrations for Group 1 was 4.12 ± 1.1, 4.04 ± 1.5, and 3.35 ± 1.8 ng/mL for the saline, KI, and NH_4_ClO_4_ treatments, respectively. No statistical significance was determined between treatment groups. Serum TSH concentrations were below the limit of detection of the assay (1.4 ng/mL) in Group 2. 

## 4. Discussion

The objective of this study was to compare the relative efficacy of stable iodide and perchlorate to purge ^131^I^−^ from the body of rats. We evaluated the efficacy of these post-exposure treatments by monitoring ^131^I^−^ in the thyroid gland, urine, and serum for up to 3 days after dosing. This study design was based on a previous study in our laboratory [15], which showed that at 15 hours after treatment with KI or NH_4_ClO_4_, the perchlorate treated rats excreted three times the amount of ^131^I^−^ in urine compared with control treatments, while stable iodide treated rats excreted only twice the amount of ^131^I^−^ in urine compared with control treatments. In the current study, we confirm our earlier results that perchlorate treatment led to faster ^131^I^−^ excretion compared with KI treatment (Table 2). Interestingly, by 75 hours after dosing there was no difference in the total amount of ^131^I^−^ excreted in urine between treatment groups and the controls. This attenuation of the initial efficacy can be attributed to the short half-lives of perchlorate and iodide, 7.3 and 6 hours respectively [17,24], and suggests that repeated dosing with either treatment may most effectively clear ^131^I^−^ from the human body and thus protect the thyroid from radiation poisoning. 

One interesting finding in this study is that rats treated with T_4_ retained more thyroidal ^131^I^−^ than rats without T_4_ treatment for all treatment groups at 75 hours after dosing (Figure 4). Other metrics of ^131^I^−^ internal exposure for urine and serum were comparable for Groups 1 and 2, *i.e*., no significant differences were determined between groups in the cumulative amounts of ^131^I^−^, stable iodide, or perchlorate excreted in the urine at 75 hours after dosing or in the concentrations of ^131^I^−^, stable iodide, or perchlorate in serum at 15 and 75 hours after dosing. Although the goal of T_4_ administration was to inhibit spikes in serum TSH levels after administration of KI or NH_4_ClO_4_, the outcome was diminished serum TSH levels below euthyroid levels, thus altering the thyroid gland function. The stimulatory effect of the reduced levels of TSH in T_4_-treated rats on thyroid hormone secretion was probably very low compared with the non-T_4_ treated rats, resulting in increased residual ^131^I^−^ radioactivity. The increase in residual thyroid radioactivity can likely not be attributed to the well known Wolff-Chaikoff effect given its transient nature relative to the duration of the experiment. However, the Wolff-Chaikoff effect must be considered if multiple doses of the treatments are administered over time. 

The effectiveness of stable iodide and perchlorate as radioprotectants for ^131^I^−^ uptake into the thyroid gland is time-dependent [25,26]. Zanzonico and Becker (2000) [25] determined that when KI is administered to rats 2 hours after ^131^I^−^ exposure there is an 80% reduction in uptake of radioactive iodide in the thyroid. However, when KI is administered 8 hours after ^131^I^−^ exposure the reduction in uptake of radioactive iodide is reduced by 50%. Sinadinovic and Jovanovic (1971) [26] concluded that when rats were administered perchlorate and KI 30 min or 24 hours prior to radioiodide tracer administration, the perchlorate treated animals accelerated the elimination of radioiodide from the body, reducing its systemic biological half life, compared to KI treated rats. In our experiments, the KI treated rats excreted the same total amount of radioiodide at 3 days post exposure as perchlorate treated animals, but at a slower excretion rate. We plan further experiments to evaluate the efficacy of repeated treatments of perchlorate and KI.

In conclusion, our findings suggest that stable iodide and perchlorate appear equally potent at enhancing urinary elimination of radioiodide over a 3 day period. However, the perchlorate treatment offers an accelerated elimination rate of radioiodide within the first 24 to 36 hours and has a significant reduction in the biological half-life of ^131^I^−^ in the first 24 hours. This suggests that repeated doses of perchlorate post radioiodide exposure may increase the effectiveness of blocking uptake in the thyroid and eliminating radiation in the urine. These data support continued consideration of perchlorate as a prophylactic drug to treat radioiodide poisoning. Intentional and unintentional releases of radiation remain a public health concern in many countries and more research is needed to identify the most effective therapies for protecting the thyroid from radioiodide poisoning. 

## Disclaimer

The findings and conclusions in this report are those of the author(s) and do not necessarily represent the official position of the Centers for Disease Control and Prevention/the Agency for Toxic Substances and Disease Registry or the U.S. Food and Drug Administration. 
